# Detecting microRNA activity from gene expression data

**DOI:** 10.1186/1471-2105-11-257

**Published:** 2010-05-18

**Authors:** Stephen F Madden, Susan B Carpenter, Ian B Jeffery, Harry Björkbacka, Katherine A Fitzgerald, Luke A O'Neill, Desmond G Higgins

**Affiliations:** 1 School of Medicine and Medical Science, Conway Institute, University College Dublin, Belfield, Dublin 4, Ireland; 2 School of Biochemistry and Immunology, Trinity College Dublin, Dublin 2, Ireland; 3 National Institute for Cellular Biotechnology, Dublin City University, Glasnevin, Dublin 9, Ireland; 4 Department of Clinical Sciences, Malmö University Hospital, Lund University, Malmö, Sweden

## Abstract

**Background:**

MicroRNAs (miRNAs) are non-coding RNAs that regulate gene expression by binding to the messenger RNA (mRNA) of protein coding genes. They control gene expression by either inhibiting translation or inducing mRNA degradation. A number of computational techniques have been developed to identify the targets of miRNAs. In this study we used predicted miRNA-gene interactions to analyse mRNA gene expression microarray data to predict miRNAs associated with particular diseases or conditions.

**Results:**

Here we combine correspondence analysis, between group analysis and co-inertia analysis (CIA) to determine which miRNAs are associated with differences in gene expression levels in microarray data sets. Using a database of miRNA target predictions from TargetScan, TargetScanS, PicTar4way PicTar5way, and miRanda and combining these data with gene expression levels from sets of microarrays, this method produces a ranked list of miRNAs associated with a specified split in samples. We applied this to three different microarray datasets, a papillary thyroid carcinoma dataset, an in-house dataset of lipopolysaccharide treated mouse macrophages, and a multi-tissue dataset. In each case we were able to identified miRNAs of biological importance.

**Conclusions:**

We describe a technique to integrate gene expression data and miRNA target predictions from multiple sources.

## Background

MicroRNAs (miRNAs) are non-coding RNAs of approximately 22 nucleotides (nt) in length that regulate gene expression through translational inhibition or mRNA degradation [1,2]. MiRNAs have been shown to play an important role in a wide variety of biological processes such as apoptosis [3], cell proliferation [4] and carcinogenesis [5]. Currently there are approximately 10,000 miRNAs from 115 species in miRBase, an online database and repository for miRNAs [6].

Computational miRNA target prediction is a key component in predicting miRNA action. Although miRNAs are ~22nt in length, it has been shown that the ~6nt 5' miRNA 'seed' region is the most crucial component for recognising and binding to target sites in the 3'UTRs of genes [7]. Most miRNA target prediction programs exploit this complementarity as well as the fact that true sites tend to be conserved between related species. TargetScan, PicTar and miRanda all use cross species conservation and different ways of measuring seed complementarity in their prediction algorithms [8-11]. More recently there have been several prediction methods based on filtering or processing the above databases e.g. MiRTif [12] and NBmiRTar [13].

Gene expression microarrays are widely used to measure mRNA gene expression levels in biological material. When differences are observed between two conditions or between and experiment and a control, most of these differences are likely to be due to differences in transcriptional activity. Some differences, however, may also be due to the actions of miRNAs. Clearly, if a miRNA acts through translational repression, then you do not expect to see this reflected in differences in the mRNA levels of its targets. However, the effects of miRNA directed mRNA degradation may be detectable through changes in the expression of miRNA target genes. This has been exploited recently to analyse mRNA gene expression datasets to predict miRNA activity. The basic principle is to search for overrepresentation of miRNA target sites in sets of genes which are down regulated [14,15]. In each case they linked gene expression data and miRNA target predictions.

Arora and Simpson [14] used a combination of three different statistical tests to detect miRNA signatures from gene expression data, the wilcoxon rank sum test, the 'rank ratio test' [16], and the absolute expression t-test. They used these tests to identify tissue specific miRNAs in both human and mouse, based primarily around TargetScan predictions. Cheng and Li [15], use an enrichment score, where a ranked vector of genes is compared to a ranked vector of degenerated binding score profiles in which miRNA target prediction binding scores (from miRanda [11]), above and below a certain threshold are set to 1 and 0 respectively. This is similar to the gene set enrichment algorithm (GSEA) [17]. They identified the activity enhancement of miRNAs that were transfected into HeLa cells and showed that their method performed better then GSEA and the wilcoxon test.

In this paper we describe the use of a multivariate statistical technique called co-inertia analysis (CIA) [18,19] that can be used to link gene expression data and miRNA target predictions from multiple programs to associate miRNAs with particular diseases or conditions. This is a simple yet highly effective approach that allows us to simultaneously analysis whole microarray datasets and databases of miRNA target predictions, and visualise the data in linked two dimensional plots. This allows us to visually identify miRNAs that are associated with particular groups in the data. The analysis can be supervised using a discriminant technique called Between Groups Analysis (BGA) [20] to focus on groups of arrays that are of a priori interest. This approach is especially useful as there is no requirement for the filtering of gene expression data or the generation of gene lists or clusters. The method can take an entire microarray dataset and cross reference/integrate it with miRNA prediction databases without the use of user defined thresholds. CIA can be used in a supervised mode where we specify groups in advance. It can also be used for data exploration in an unsupervised mode. This is useful in cases where the samples show great heterogeneity or are poorly characterised, as happens, for example, in many cancer related datasets.

In this paper we use CIA to predict miRNA activity in three different gene expression microarray datasets. The first is a papillary thyroid carcinoma (PTC) dataset [21], where both mRNA microarray and miRNA expression data for the same tissues, were available. This allows us to compare our predicted miRNAs against those that are actually observed to be highly expressed in the tissue. The second is an in-house dataset where we measured gene expression in mouse macrophages from wild type versus MAL knockout mice, after treatment with lipopolysaccharide. The results were confirmed by directly measuring the levels of predicted miRNAs, using RT-PCR. The third dataset was that used by Arora and Simpson [14] to demonstrate their miRNA prediction method.

## Results

For each dataset we used CIA to simultaneously analyse mRNA gene expression data and predicted miRNA target sites in the 3' UTRs of the same genes. The starting point is two tables: one table of gene expression values for *g *genes from *n *"samples" (*n *microarrays) and one table giving the counts of predicted target sites for m miRNAs in the same *g *genes. These tables (*gxn *and *gxm*) are analysed using Non-symmetric Correspondence Analysis (NSC) and linked using CIA. The CIA analysis gives us diagrams which can be visually inspected and show the relationships between gene expression differences in different "samples" and how these relate to differences in the occurrence of miRNA target sites. This is unsupervised and is used for data exploration and visualisation purposes.

The analysis can be made supervised by applying Between Group Analysis (BGA, [18,22]) which takes class information and a NSC analysis and finds axes or vectors that best discriminate pre-assigned groups by maximising the between group variance. We use this technique to automate the analysis by specifying a predetermined split in the microarray samples such as between those from normal and cancer tissue, and so identify putative regulating miRNAs associated with the split. The result of a BGA analysis on 2 groups is a ranked list of miRNAs.

### Papillary Thyroid Cancer Dataset

**Figure 1:**
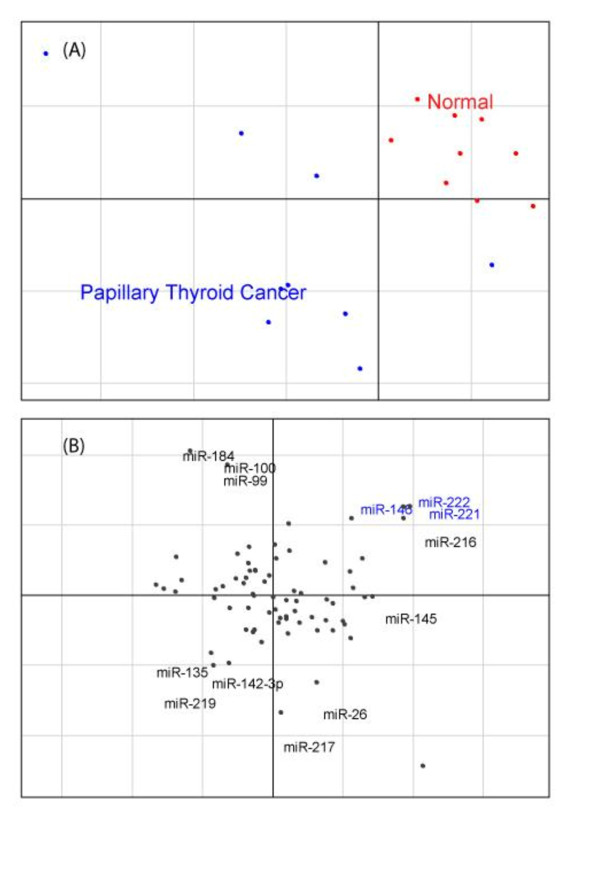
**Unsupervised CIA of the papillary thyroid carcinoma dataset**. Axis 1 (horizontal) and 2 (vertical) of the unsupervised CIA for the PTC dataset. The gene/miRNA frequency table generated from TargetScanS was used to make the figure. (A) Shows the projection of the PTC (blue) and normal thyroid tissue (red). The first and second axes split the data with the more homogeneous normal tissue samples clustering in the top right hand corner. (B) Shows the projection of the miRNAs. MiRNAs that are in the opposite orientation relative to the origin are associated with those samples. Highlighted in blue are miRNA associated with PTC identified on the miRNA microarray.

We first applied CIA to find miRNAs associated with papillary thyroid cancer (PTC). He et al [21] produced mRNA and miRNA gene expression data using microarrays from PTC and adjacent unaffected tissue for 9 patients. CIA was applied to the 18 microarrays (9 PCT and 9 unaffected tissue), and the associated miRNA/gene frequency tables. To simplify the comparison between our prediction technique and the experimental data, only those miRNAs present on the miRNA microarray, were analysed (see additional file 1: 'MiRNAs on the OSU_CCC version 2.0 miRNA microarray chip' for details). The full analysis including all miRNAs predicted by the target prediction software are available in additional file 2: 'Results of the CIA for PTC using all available miRNAs'. This includes miRNAs that were not tested by He et al. [21] but may be potential novel miRNAs involved in PTC. Axes 1 and 2 of the resultant CIA for the gene expression microarrays using the TargetScan gene/miRNA frequency table can be seen in figure 1.

In figure 1a we see the PTC and the normal thyroid tissues highlighted in blue and red respectively. As you would expect the normal samples are more homogeneous, than the PTC samples. Figure 1b, shows the miRNA motifs associated with this split in the data. The most extreme motifs along each axis are labelled and named. Motifs that are in the opposite orientation (direction from the origin) as the PTC array samples are associated with PTC. This is counterintuitive, but reflects the way in which miRNAs usually negatively regulate gene expression. Genes that are upregulated in normal tissue may contain predicted binding sites for miRNAs upregulated in PTC. In other words, these genes are subsequently down regulated in the presence of these miRNAs. Those miRNAs highlighted in blue (miR-221, miR-222, and miR-146) are predicted to be upregulated in PTC, from visual inspection of the plots.

In order to systematically identify the miRNAs specifically associated with the split we are interested in (9 PTC vs 9 normal tissue), we performed a supervised analysis of the data, combining CIA and BGA. Informally this is the equivalent of plotting a line between the centres of the two groups in figure 1a and then finding the equivalent line in the lower panel (with the same orientation from the origin) and plotting the miRNA motifs along this. This is done automatically by BGA and produces a single vector with the co-ordinate of every miRNA. This procedure was repeated for each of the 5 miRNA/gene frequency tables, one for each of the 5 miRNA prediction programs. This returns 5 lists of miRNA motifs that are ranked based on the motifs association with PTC. As each of these programs has distinct characteristics, they returned different lists of motifs. To allow for this, we only considered those motifs which ranked highly (in the top 20) with two or more of the programs.

Table 1 shows the results of the comparison between PTC and normal thyroid tissue. It also contains the ranking of each miRNA for each of the 5 programs and the average ranking. The miRNAs that were identified by He et al. [21] are highlighted in bold. Although we did not predict all miRNAs that were upregulated in PTC, we did identify the four mostly highly upregulated, based on their analysis, miR-146, miR-221, miR-222, miR-21 (upregulated 19.3 fold, 12.3 fold, 10.9 fold and 4.3 fold respectively) (Table 1). MiR-146, miR-221 and miR-222, are an order of magnitude more upregulated than any of the other miRNAs.

**Table 1:** miRNAs predicted to be associated with papillary thyroid cancer

Predicted miRNAs	Rank with PicTar4way	Rank with PicTar5way	Rank with TargetScan	Rank with TargetScanS	Rank with miRanda	Average Rank	Fold Change
**miR-222**	3	2	3	4		3	10.9
**miR-221**	6	4	2	3		3.75	12.3
miR-346	4		5			4.5	-
miR-142	7	3	10		2	5.25	-
**miR-146**				5	14	9.5	19.3
miR-144				13	6	9.5	-
miR-134	14		6			10	-
miR-183	13	16	9	6		11	-
miR-126*	20				3	12.5	-
miR-1				16	10	13	-
miR-206				17	11	14	-
miR-200a/b/c			18		12	15	-
**miR-21**	18			16		17	4.3
miR-223	19			17		18	-
miR-100				20	18	19	-

The miRNAs which are also identified by the miRNA microarray are highlighted in bold

**Figure 2:**
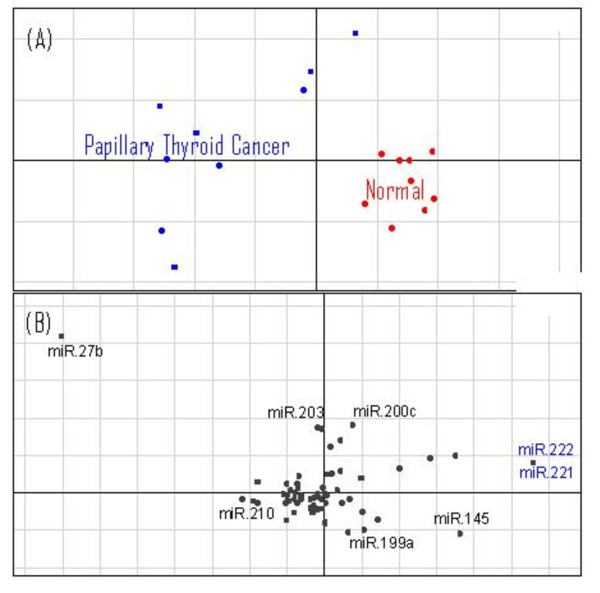
**Axes 1 and 2 of the CIA of the PTC dataset using miRecords target data**. Unsupervised CIA of the PTC dataset generated using the gene/miRNA frequency table from miRecords target data. (A) Shows the projection of the PTC (blue) and normal thyroid tissue (red). Axis 1 (horizontal) splits normal thyroid tissue samples from the PTC samples. (B) Shows the projection of the miRNAs. MiRNAs that are in the opposite orientation relative to the origin are associated with those samples. Highlighted in blue are miR-221 and miR-222, which are associated with PTC.

The above analysis was performed using computationally predicted target sites. These predictions are noisy with a high false positive and false negative rate. In order to test our approach with experimentally verified miRNA targets we also applied CIA to data from miRecords [23].This is a resource which contains relatively small numbers of high quality experimentally confirmed miRNA targets. MiRecords currently contains target information for 90 miRNAs across 599 human genes. Although this is not the only database for experimentally verified miRNA targets it is comprehensive, well curated and comparable to other databases such as Tarbase [24]. Again this was used to find miRNAs associated with PTC. Axes 1 and 2 of the resultant CIA can be seen in figure 2. In figure 2a we can see a plot of the 18 samples. The plot is similar to figure 1a in that the more homogeneous normal samples are clustered together (red), while the PTC samples (blue) are more scattered. Figure 2b shows the miRNAs associated with the split in the data. To the right of the figure, along the horizontal axis, we can see miR-221 and miR-222 highlighted in blue. Again these motifs are in the opposite orientation relative to the origin of the PTC samples in figure 2a, and are predicted to be upregulated in PTC. Performing a supervised analysis on this dataset using BGA confirms that miR-221 and miR-222 are the miRNAs most highly associated with PTC.

This analysis confirms that CIA is capable of integrating both predicted and experimentally confirmed miRNA target information with gene expression data. Ideally it would be preferable to focus on experimental rather than predicted miRNA data, but at present this is not feasible as currently available miRNA target data are limited. For example, there are no confirmed targets of miR-146 in miRecords. This is the most highly upregulated miRNA in the PTC data set, and therefore we would only be able to detect this miRNA using target predictions.

### LPS Treated Macrophages Dataset

In this dataset, bone marrow derived macrophages from wild type (WT) and MAL knockout (MALKO) mice were treated with lipopolysaccharide (LPS). MAL (MyD88-adaptor-like) is part of the Toll-like receptor (TLR) signalling pathway, a key signalling molecule of the innate immune response [25]. The innate immune response is triggered in the presence of the Gram-negative bacterial product LPS. MAL-deficient mice are known to be defective in TLR2 and TLR4 signalling but show normal signalling with other members of the TLR family [26]. The aim of this experiment was the identification of miRNAs whose activation is independent of MAL i.e. is upregulated in both MALKO and WT macrophages after exposure to LPS. In total there were four replicates of LPS treated WT arrays, and four replicates of LPS treated MALKO arrays.

**Figure 3:**
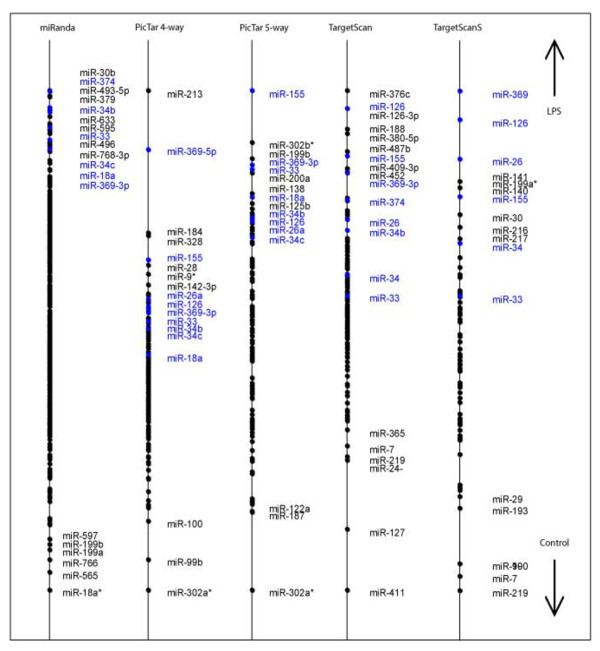
**Supervised CIA results for the LPS treated WT macrophages**. Supervised analysis using BGA was performed with the gene expression data and the 5 gene/miRNA frequency tables. Each axis shows the projection of the miRNAs that are predicted to be upregulated in LPS treated WT macrophages produced with each of the 5 target prediction programs. Highlighted in blue are miRNAs that are highly ranked by multiple programs.

**Figure 4:**
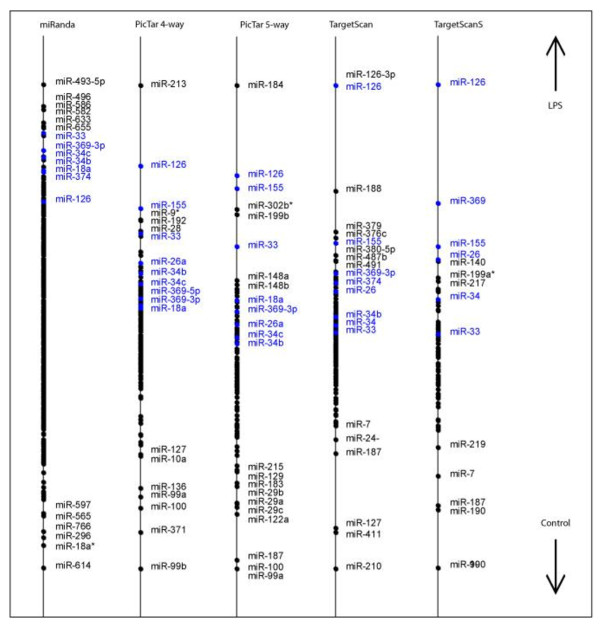
**Supervised CIA results for the LPS treated MALKO macrophages**. BGA was performed with the gene expression data and the 5 gene/miRNA frequency tables. Each axis shows the projection of the miRNAs that are predicted to be upregulated in LPS treated MALKO macrophages. The axes were produced with the 5 target prediction programs. Highlighted in blue are miRNAs that are highly ranked by multiple programs.

**Figure 5:**
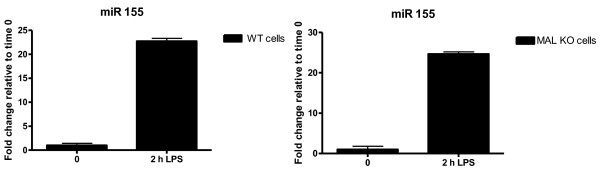
**miR155 is induced following LPS stimulation**. Bone marrow derived macrophages (BMDM) were obtained from wild-type (WT) and Mal knockout mice. Cells were differentiated for 10 days and stimulated with 100 ng/ml LPS for 2 h. RNA was extracted and miR155 levels were tested by quantitative RT-PCR. Results are expressed as a mean ± S.D for triplicate determinations. All results are representative of 3 separate experiments.

**Figure 6:**
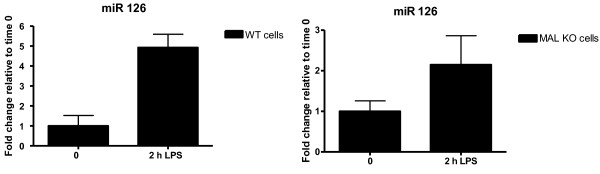
**miR126 is induced following LPS stimulation**. Bone marrow derived macrophages (BMDM) were obtained from wild-type (WT) and Mal knockout mice. Cells were differentiated for 10 days and stimulated with 100 ng/ml LPS for 2 h. RNA was extracted and miR126 levels were tested by quantitative RT-PCR. Results are expressed as a mean ± S.D for triplicate determinations. All results are representative of 2 separate experiments.

As with the thyroid cancer dataset, supervised CIA was performed using BGA for each of the 5 gene/miRNA frequency tables, on the two datasets to identify MAL-independent miRNAs. This returns 5 lists of miRNAs that are ranked based on their association with LPS treated cells. The lists vary depending on the individual characteristics of the prediction programs used. The resulting axes are shown in figure 3 for the WT dataset and figure 4 for the MALKO dataset. The motifs are ranked based on their association with LPS treated macrophages. These plots illustrate the overlap between the WT and the MALKO results i.e. those miRNAs that are MAL independent. They also demonstrate the variation between the different miRNA target prediction programs. Again we selected those motifs which ranked highly (in the top 20) with two or more of the programs, as being most likely to be true results. The results can be seen in table 2 for both datasets and are highlighted in blue in figures 3 and 4. Table two only contains those miRNAs that are MAL independent, and gives the ranking for each of these miRNAs. From this table miR-155 and miR-126 were selected for experimental confirmation of their induction by LPS using quantitative RT-PCR. These miRNAs were chosen as they were predicted to be upregulated in both MALKO and WT by four of the programs, and they had a very low average ranking in the two datasets. The consistently low ranking across all four programs in both MALKO and WT cells suggested that these predictions were the most likely to be correct and were therefore chosen as the most suitable miRNAs for further experimental confirmation.. In figure 5 we can see that miR-155 is 23 fold and 25 fold upregulated in WT and MALKO LPS treated macrophages respectively. This is based on the expression of miR-155 relative to time zero, in macrophages extracted from MAL-deficient mice, after being treated with LPS for two hours. MiR-155 has been previously shown to be an LPS inducible miRNA [27], and this analysis confirms that its induction is also MAL independent. Figure 6 shows that miR-126 is 5 fold and 2 fold upregulated in WT and MALKO macrophages respectively. The upregulation of the miRNA in this case is not as strong as with miR-155 but is still consistent with the hypothesis that this miRNA is MAL independent.

**Table 2:** miRNAs predicted to be up-regulated in Macrophage cells treated with LPS

Predicted miRNAs	Rank with PicTar4way	Rank with PicTar5way	Rank with TargetScan	Rank with TargetScanS	Rank with miRanda	Average rank
*WT macrophages*						
miR-369/3p/5p	2	4	10	1		4.25
**miR-155**	**5**	**1**	**7**	**7**		**5**
miR-374			12		2	7
**miR-126**	**15**	**14**	**2**	**2**		**8.25**
miR-34/b/c		15		12	5	10.66
miR-33	20	5			8	11
miR-26/a	11	16	18	3		11.25
miR-18a		9			20	14.5
						
*MALKO macrophages*						
**miR-126**	**2**	**2**	**1**	**1**		**1.5**
**miR-155**	**3**	**3**	**6**	**3**		**3.75**
miR-33	8	6			7	7
miR-26/a	13			4		8.5
miR-369/3p/5p		20	11	2	11	11
miR-34/b/c	19			13	14	15.33
miR-18a		15			18	16.5
miR-374			18		20	19

Experimentally verified miRNAs are in bold

### Comparison with Arora and Simpson

Previously Arora and Simpson developed an approach to detect the effect of particular miRNAs on their target mRNA gene expression levels [14]. As with our approach, they used gene expression data and publicly available miRNA target prediction data. Their analyses were based primarily around TargetScan target predictions (version 3.1). They used three statistical tests to detect miRNA signatures from gene expression data, the wilcoxon rank sum test, the 'rank ratio test' [16], and the absolute expression t-test. They used these techniques for the identification of tissue specific miRNAs, using data from a number of sources including GEO series GSE3256 [28-30]. This dataset comprised 353 microarrays spanning 65 normal human tissues. Arora and Simpson focused on a comparison of 8 tissue types using one representative microarray from each tissue type (there were multiple microarrays for each tissue type). Extensive literature mining was used to identify evidence of tissue specific expression profiles of the miRNAs. This evidence included cloning, Northern hybridization and expressed sequence tag mapping [14].

**Figure 7:**
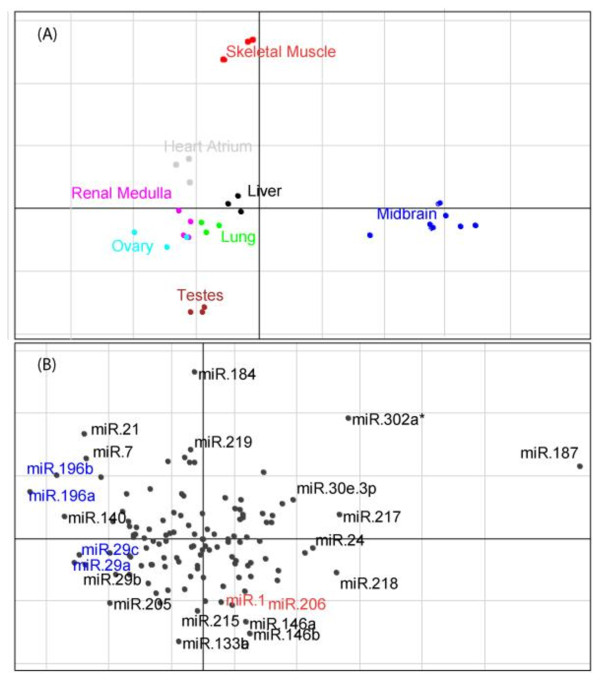
**Axes 1 and 2 of the unsupervised CIA for the tissue dataset**. The gene/miRNA frequency table generated from PicTar5way was used to make this figure. (A) Shows the projection of the tissue samples. Axis 1 (horizontal), separates the midbrain tissue (blue) from the rest of the samples, while axis 2 (vertical) separates skeletal muscle tissue (red) and to a lesser extent heart tissue (grey) from the rest of the samples. (B) Shows the projection of the miRNAs. Motifs in the opposite orientation relative to the origin are associated with that tissue. Highlighted in blue are miRNA associated with brain tissue, and in red are motifs associated with skeletal muscle.

We applied unsupervised CIA to the same 8 tissues used by Arora and Simpson [14]: midbrain, heart atrium, kidney medulla, liver, lung, ovary, skeletal muscle, and testis. In total there were 29 microarrays, as we included all microarrays from each tissue in our analysis rather than one representative microarray. These were combined with the 5 miRNA/gene frequency tables in the unsupervised CIA. Axes 1 and 2 of the resultant CIA for the PicTar5way table can be seen in figure 7a. Visual inspection of this plot reveals a number of interesting splits in the data.

In figure 7a, the skeletal muscle (red) is separated from the other tissues along the vertical axis. Figure 7b, shows the miRNAs associated with each tissue type. As with the PTC, it is the motifs that are in the opposite orientation (direction from the origin) as the skeletal muscle, that are associated with skeletal muscle. This can be seen in the case of miR-1/206 (highlighted in red) located in the centre bottom of figure 7b. This is in the opposite orientation relative to the origin and is a known muscle specific miRNA [31]. This miRNA was not identified by Arora and Simpson as being associated with skeletal muscle, but was associated with heart tissue (grey in figure 7a). Another major split in this data, is that of the midbrain tissue (blue) along the horizontal axis. Again it is the miRNAs in figure 7b that are in the opposite direction relative to the origin that are associated with brain tissue. These include miR-196a/b and miR-29a/c. These miRNAs were also identified by Arora and Simpson, although only miR-29a/c has been previously characterised in brain tissue [32,33].

In order to systematically identify tissue specific miRNAs we compared individual tissues against the rest e.g. the 3 skeletal muscle microarrays vs. the rest. As before, this supervised CIA was performed using BGA, and consistency between miRNA prediction programs was required. Table 3 summaries the miRNAs we identified, and the overlap between our analysis and that of Arora and Simpson, as well as information on available experimental data. As can be seen from the table, there is a great deal of overlap between the two datasets. For example, a known muscle specific miRNA, miR-1/206, was identified by both techniques in the heart. However, only our technique identified miR-1/206 in skeletal muscle, while we did not identify brain specific miR-124. A direct comparison between the two approaches is difficult as we used more recent miRNA target predictions and a greater number of microarrays were tested (we used more than just a representative microarray for each tissue). However, approximately a quarter of the predictions overlap between the two approaches with our approach identifying approximately 15% less miRNAs. Also for both of the techniques roughly half the results are supported by literature review (~44% vs 47%). This suggests that although neither approach is exhaustive they are complementary.

**Table 3:** Overlap between predicted tissue specific miRNAs, Arora and Simpson data, and available experimental data

Predicted miRNA	Brain	Heart	Kidney	Liver	Lung	Ovary	Skeletal Muscle	Testes
let-7/a/b/f	Y*, 1,2,3	*	*	*	*	*	*	*
miR-1/206		Y*, 1,2,3		*			Y, 2	
miR-7	Y, 2		Y					
miR-10/a/b			*	Y, 10		*		
miR-15/a/b	*		Y*, 15		*	Y*, 18		*
miR-18/a/b								Y
miR-21	Y, 4			Y, 10				
miR-22		Y, 3			Y		Y	
miR-23a/b					Y, 2			
miR-24/*		*			*	Y*, 18		
miR-29/a/b/c	Y*, 2,3		*					
miR-30	*	*	Y*, 2,15	*	*	Y*, 18	*	*
miR-33					Y		*	*
miR-34/b/c						*	Y	Y*, 16
miR-99/b		Y, 1	Y					
miR-101	*			Y*, 2			*	
miR-122/a				Y*, 1,2,3				
miR-125/a/b	*	Y*, 1,2	*		*	Y*, 18		
miR-126			Y, 2		Y, 2			
miR-127		Y					Y	
miR-128/b	*		*		*	*	Y*	
miR-129/3p							Y	
miR-133/a/b		Y*, 2,3					Y*, 2,3	
miR-134		Y						
miR-135/a/b			Y			Y		
miR-136					Y			*
miR-137	*			Y*	Y*	*		
miR-141			Y, 8		Y			
miR-142-3p/5p		Y			Y, 11			Y
miR-143	Y, 3							
miR-146/a/b	Y, 1			Y	Y*, 12	*		
miR-151					Y			
**miR-152**				**Y**				**Y**
miR-181a						Y		
miR-182/*				Y		Y		Y
miR-184	Y, 17							
miR-187								Y
miR-188						Y		
miR-190			*		Y			
miR-191				Y				
**miR-195**						**Y, 18**		
miR-196/a/b	Y*	*	*			*	*	*
miR-199/b	Y, 5	Y, 15					*	
miR-200/a/b/c			Y, 7,8	Y, 10			Y	
miR-205								Y
miR-208				Y				
miR-210				Y				Y
**miR-212**				**Y**				
**miR-215**								**Y**
miR-217					Y	Y		
miR-219	Y, 1					Y		
miR-221					Y, 13			Y
miR-222				Y, 10				Y
miR-223			Y, 9					
miR-299-3p/5p	Y							
**miR-302**				**Y**				
miR-324-5p					Y			
miR-325			Y		Y	Y		
miR-326						Y		
miR-335	Y, 4							*
miR-340	Y							
miR-361			Y					
miR-369-3p/5p			Y					
miR-375	Y*							
miR-411			Y					

Y represents miRNAs predicted by CIA. * represents those miRNAs also predicted by Arora and Simpson. Those miRNAs in bold were not incorporated into TargetScan version 3.1 (used by Arora and Simpson). The numbers are the references from available experimental data. References: 1. Gu et al., [44], 2. Sempere et al., [33], 3. Lagos-Quint et al., [32], 4. Sathyan et al., [45], 5. Hua et al., [46], 6. Chan et al., [47], 7. Gregory et al., [48], 8. Nakada et al., [49], 9. Anglicheau et al., [50], 10. Ladeiro et al., [51], 11. Lui et al., [52], 12. Raponi et al., [53] 13. Navarro et al., [54], 14.Lagos-Quint et al., [55], 15. Naraba et al., [56], 16. Yu et al., [57], 17. Nomura et al., [58], 18. Baskerville et al. [35].

## Discussion

In this paper we describe a method for inferring the action of miRNAs by integrating the information provided by miRNA target prediction programs with mRNA gene expression data. This method can be used to predict the activity of a miRNA when there is down regulation of multiple potential target genes for that miRNA.

A drawback of this and similar approaches is that it assumes that mRNA degradation instead of translation repression is the primary mode of action of the miRNAs. This will only be true for some miRNAs. MiRNAs which act through repression will be undetectable using our methodology. Instead, the action of the miRNA might be inferred through correlating genomic locus with co-expression of nearby genes [34,35].

A further drawback of the current method is that it relies on predictions of target sites in potential target mRNAs. Such predictions will contain false positives as well as false negatives and it is often difficult to strike a balance between sensitivity and specificity. Nonetheless, we have shown the current method does provide good quality predictions of miRNA activity. In the case of PTC, the method was able to predict miRNAs which had been experimentally measured as showing significant differential expression. In the case of the MAL knockout data set, we were able to verify predicted miRNAs using RT-PCR. In the third dataset, we obtained comparable results to the method of Arora and Simpson [14] whose method has already been partly validated, experimentally.

A major advantage of this method is that no pre-processing of gene expression data, such as the generation of clusters or gene lists, is required. This is in marked contrast to most other methods such as those based on Gene Set Enrichment Analysis (GSEA) [17]. The method can take an entire microarray data set and cross reference/integrate it with miRNA prediction databases. We also use multiple miRNA target gene predictions. This allows us to use consistency across target prediction programs and so minimise the effect of weaknesses in any one program, while maximising the number of miRNAs that can be investigated. The congruence between the sets of predicted targets of miRNA sets can often be weak, particularly between TargetScan/PicTar vs. miRanda. Even the confirmed targets from miRecords [23] may often be context specific due to tissue specific expression and 3' UTR splice variants. Methods in the past have often used just one of the predicted databases [14,15] and so it may be assumed the results would vary depending on the choice of miRNA target prediction software used. By using multiple miRNA target gene predictions we can use consistency across target prediction programs to maximise the reproducibility of the analysis, while retaining a large proportion of the miRNAs. Creighton et al [36] also developed a technique that facilitated the use of multiple target prediction algorithms, to associate miRNAs with gene expression profiles of interest. However, they required the user to specify a predefined list of genes, while our approach does not require any pre-processing of gene expression data.

The method can also be used to visualise and analyse multiple groups of arrays. In the data sets that we tested in this paper, we only compared two groups at a time, in order to compare the method with other methods. The visualisations are powerful as they allow simple graphical representations of highly complex datasets and the relationships between them.

## Conclusions

In this study we have shown how CIA can be used to integrate gene expression data and miRNA target prediction data from multiple sources to predict miRNAs associated with particular diseases or conditions. Although this is not the first attempt to predict miRNA expression profiles using gene expression data, we believe that it is complementary to other currently available methods, and would be a useful addition to the field. The method allows clear visualisation and data exploration of complex datasets. It can be used in a supervised or un-supervised mode and can detect the activity of miRNAs which have been experimentally measured as being expressed.

## Methods

### Co-inertia analysis

To study two linked data tables simultaneously, we used CIA, a multivariate coupling approach that was first introduced to study ecological data [18,19]. In this case the two linked tables are expression data, and a miRNA frequency table on the same set of genes. We treat these as two sets of measurements on the same objects, the genes. We have previously used this method to compare gene expression data with transcription factor binding site information [37], and proteomics data [38]. CIA is used in conjunction with an ordination method such as non-symmetric correspondence analysis (NSC) or principal components analysis (PCA). These methods summarise a data table in a low dimensional space, by projecting the samples onto axes which maximise the variances of the coordinates of the projected points. CIA performs two simultaneous NSCs on the two linked tables, and identifies pairs of axes, from the two datasets which are maximally covariant [37].

BGA is a supervised classification method which can be used in combination with ordination methods, which forces an ordination to be carried out on groups of samples rather than individual samples [20,22]. First a normal NSC is performed, BGA then finds the linear combination of the NSC axes that maximizes between-group variance and minimizes within-group variance, for specified groups.

BGA can be used to perform a supervised NSC on the gene expression data by directing the CIA to find the maximum co-variance between the gene expression difference between groups of samples and the miRNA-gene target frequency tables. We have two data tables. One table gives genes by target sites and the second genes by gene expression data. Two simultaneous NSCs are performed on the two tables using BGA. We find two resultant axes, one for each dataset with minimal between group variance. This forces the analysis to rank arrays or tissues along a first axis that best discriminates the two groups of samples (e.g. PTC versus normal thyroid tissue) and a second axis with ranked miRNAs. The two axes are found as the ones that maximize their covariance. The miRNAs that are upregulated in a group of samples are those that are at the opposite end of the miRNA axis to those samples. For example, genes upregulated in normal thyroid, contain binding sites for miRNAs that are upregulated in PTC (e.g. miR-221). Therefore miR-221 is at the opposite end of the miRNA axes to PTC on the sample axes. This is indicative of the decrease in gene expression caused by the miRNA that we are attempting to identify. Thus, for each split in the data that we specify using BGA, we get a ranked list of miRNA motifs.

We get a separate ranked list of motifs for each of the miRNA prediction methods used. We used five prediction methods (see below). The results are combined using consistency among the prediction programs. To be considered a true result a motif was required to be highly ranked (top 20) by two or more programs. This produces a single ranked list of miRNAs expressed in a particular disease or condition. All calculations were carried out using the MADE4 library [39] of the open source R package. MADE4 can be downloaded freely from the Bioconductor web site http://www.bioconductor.org. All the scripts used are available upon request from the authors.

### MiRNA target prediction

Five different miRNA target prediction programs were used, TargetScan and TargetScanS [8,9], PicTar4way and Pictar5way [10], and miRanda [11]. Each of these programs search for complementarity to the miRNA seed region in the 3'UTRs of mRNAs and incorporate cross species conservation into their target prediction calculations. The miRNA target prediction data were downloaded from the TargetScan website http://www.targetscan.org/ (version 4.1), the UCSC genome browser tract for pictar4way and pictar5way http://genome.ucsc.edu/, and from miRBase for miRanda (http://microrna.sanger.ac.uk/sequences/[6]). The overlap between the target lists varied depending on the number of miRNAs being examined and the number of predicted target genes. Two variations of the TargetScan program were used, TargetScan and TargetScanS. TargetScan predicted 7,611 target genes for 195 miRNAs, while the more stringent TargetScanS predicted 4,769 target genes for 90 miRNAs. Two version of PicTar were used, PicTar4way which incorporates conservation across 4 species and predicted 9,151 target genes for 178 miRNAs and PicTar5way which uses conservation across 5 species and predicts 3,454 target genes for 130 miRNAs. MiRanda is the least selective of the prediction programs used, predicting 17,759 target genes for 470 miRNAs. We used these data to generate a gene/miRNA frequency table of counts of predicted targets per gene, for each of the five sets of target gene predictions.

### MiRecords Data

Experimentally validated miRNA target information was downloaded from miRecords http://mirecords.biolead.org/. As with the miRNA prediction programs this information was used to construct a gene/miRNA frequency table of counts of miRNA targets per gene. In total miRecords contains data for 90 miRNAs targeting 599 genes.

### Gene Expression data

#### The Papillary Thyroid Cancer dataset

The thyroid cancer mRNA expression data were obtained from He et al. [21]. The data were downloaded from http://www.ncbi.nlm.nih.gov/projects/geo/ (Gene Expression Omnibus (GEO), accession number: GSE3467) as raw data files (.cel files). Gene expression values were called using the robust multichip average method [40] and data were quantile normalized using the Bioconductor package, affy. Affymetrix Human Genome U133 Plus 2.0 Array containing 54,675 Affymetrix probes was used. The RefSeq ids that corresponded to the Affymetrix probes were obtained using the hgu133plus2 annotation library. Probes that hit multiple genes were filtered out. If there were multiple probes for the same gene, the probes were averaged for that gene. The intersection between the target gene prediction software and the hgu133plus2 gene set was as follows, TargetScan: 5,355genes, TargetScanS: 3,311 genes, miRanda: 10,819 genes, PicTar4way: 4,781 genes, and PicTar5way: 1,693 genes. In addition gene expression information was available for 385 of the genes in miRecords.

#### The Tissue Dataset used by Arora and Simpson

Roth et al. [28] generated gene expression profiles for 65 normal adult human tissues. In total this dataset comprises 353 microarrays (GEO series GSE3526). These included 29 microarrays for the 8 tissues examined by Arora and Simpson [14] (midbrain, heart atrium, kidney medulla, liver, lung, ovary, skeletal muscle, and testis). Although Arora and Simpson only chose a representative sample for each tissue type we included all the available microarrays for each tissue type in our analysis. The tissue expression data were also downloaded from the GEO database in the form of raw data files. The GEO sample and platform accession numbers can be found in additional file 3: 'GEO sample and platform accession numbers'. The same normalisation and analysis procedure was used as for the PTC data. Affymetrix Human Genome U133 Plus 2.0 Array containing 54,675 Affymetrix probes was used. The intersection between the gene prediction software and the hgu133plus2 gene set was the same as above.

### LPS Treated Macrophage Dataset

#### Materials

MAL-deficient mice were a gift from S.Akira (Osaka, Japan) [26] and were backcrossed onto a C57BL/6 background for approximately 9 generations. LPS derived from E.Coli strain O111:b4 was purchased from Sigma, dissolved in deoxycholate, and re-extracted by phenol:chloroform as previously described [41].

#### Cell culture and RNA isolation

Mice were anaesthetized with CO_2 _inhalation and then killed by cervical dislocation. Bone marrow was isolated from 6- to 8-week-old C57BL/6 wild type and MAL-deficient mice. Macrophages from these marrows were cultured in DMEM media supplemented with 10% FBS and 15% L929 conditioned media (a source for colony-stimulating factor CSF-1) for 10 days. For mRNA expression studies by quantitative reverse transcriptase PCR (QRT-PCR) and microarray analysis in bone marrow derived macrophages (BMDMs), cells were treated at day 10 ex vivo for 2 hours with 10 ng/ml lipopolysaccharide and their gene expression profiles were compared with that of mock treated cells incubated for the same time. Cells from four individual mice for each genotype were used, and each mouse served as its own mock control. Total RNA for microarray analysis was isolated using an RNeasy extraction kit (Qiagen, Valencia, CA) according to the manufacturer's recommendations. RNA quality was assessed using the 2100 Bioanalyzer (Agilent Teachologies, Palo Alto, CA).

#### Microarray analysis

The Operon *Mus musculus *ver. 1.1 probe set (Qiagen) consisting of over 21,000 oligonucleotide probes (70-mers) was printed in the Massachusetts General Hospital (Cambridge, MA) microarray core facility using an Omnigrid 100 (GeneMachines, San Carlos, CA) on CodeLink activated slides (Amersham, Piscataway, NJ). RNA was reverse transcribed and differentially labelled with Cy3 and Cy5 dyes (Amersham) using the Atlas PowerScript fluorescent labeling kit (BD Biosciences, Palo Alto, CA). Labelled samples were hybridized overnight using an automated hybridization station (Genomic Solutions; Perkin-Elmer, Boston, MA). Fluorescent images from the arrays were acquired using a microarray scanner and its accompanying software (GenePix 4000B microarray scanner; Axon Instruments, Union City, CA). Data was stored and further quality controlled using the GeneTraffic software (Iobion Informatics, La Jolla, CA) and the BioArray Software Environment (BASE) [42]. In total there were 4 replicates for LPS treated WT cells and LPS treated MALKO cells.

The raw gene expression data (the genepix.gpr files) were read, background corrected with the "normexp" option and quantile normalised, using the Bioconductor package limma [43]. In the two datasets, the pre and post LPS treated mRNA is hybridised to the same array. This is a common experimental design for two colour arrays. For our analysis we wished to compare two groups (e.g. WT vs LPS treated WT cells), and identify which miRNAs are associated with each group using CIA. To do this it is necessary to analyse the red and green channel intensities separately i.e. as if they were two one colour arrays, and we were comparing their log-intensities rather than their log-ratios. Details for performing this analysis are available in the limma user guide [43] and relevant scripts are available upon request. The intersection between the target prediction software and the microarray gene set was as follows, TargetScan: 4,310 genes, TargetScanS: 2,879 genes, miRanda: 9,444 genes, PicTar4way: 5,205 genes, and PicTar5way: 2,000 genes.

#### RT-PCR

RT-PCR was performed using TaqMan Reverse Transcription reagents (Applied Biosystems, Foster City, CA) Kit following manufacturer's protocol and assayed on the Applied Biosystems 7900HT. The primers for miR155, miR-126 and U6 were obtained from Applied Biosystems. Data was presented as fold differences relative to time 0 and were based on calculations of 2^(-ΔΔCt)^. Ubiquitously expressed U6 small nuclear RNA was used for normalization.

## Authors' contributions

SFM assembled the data and software, carried out the analyses, analysed the results and drafted the manuscript. SBC and IBJ assisted in the analysis, the generation of figures and the drafting of the manuscript. SBC, HB, KAF preformed the microarray experiment for the LPS treated macrophage dataset and the subsequent RT-PCR confirmations. DGH and LAON conceived the project, assisted in the design of the study and in drafting of the manuscript. All authors read and approved the final manuscript.

## Supplementary Material

Additional file 1 **miRNAs on the OSU_CCC version 2.0 miRNA microarray chip**. This file contains the human microRNAs present on the miRNA microarray chip Click here for file

Additional file 2 **Results of the CIA for PTC using all available miRNAs**. This file contains the results of the CIA for PTC using all of the miRNAs. It also contains how each miRNA is ranked using the 5 different target prediction programs. Click here for file

Additional file 3 **GEO sample and platform accession numbers**. This file contains GEO accession numbers and the tissue information used to compare our results with those of Arora and Simpson. Click here for file
