# Supplementary material for: Detecting microRNA activity from gene expression data
Source: BMC Bioinformatics. 2010 May 18;11:257. doi: 10.1186/1471-2105-11-257 (PMC2885376; doi:10.1186/1471-2105-11-257)
Supplement: Additional file 2 — Results of the CIA for PTC using all available miRNAs. This file contains the results of the CIA for PTC using all of the miRNAs. It also contains how each miRNA is ranked using the 5 different target prediction programs. [file 1471-2105-11-257-S2.DOC]

Results of the CIA for PTC using all available miRNAs

| Predicted miRNAs | Rank with PicTar4way | Rank with PicTar5way | Rank with TargetScan | Rank with TargetScanS | Rank with miRanda | Average Rank | Fold Change |
| --- | --- | --- | --- | --- | --- | --- | --- |
| miR-222 | 3 | 2 | 5 | 4 |  | 3.5 | 10.9 |
| miR-221 | 6 | 4 | 4 | 3 |  | 4.25 | 12.3 |
| miR-346 | 4 |  |  | 8 |  | 6 | - |
| miR-142 | 7 | 3 | 17 |  | 3 | 7.5 | - |
| miR-126* | 20 |  |  |  | 4 | 12 | - |
| miR-134 | 14 |  | 10 |  |  | 12 | - |
| miR-183 | 13 | 17 | 16 | 6 |  | 13 | - |
| miR-144 |  |  |  | 13 | 13 | 13 | - |
| miR-21 | 18 |  |  | 16 |  | 17 | 4.3 |
| miR-223 | 19 |  |  | 17 |  | 18 | - |
|  |  |  |  |  |  |  |  |
|  |  |  |  |  |  |  |  |
