# Supplementary material for: Detecting microRNA activity from gene expression data
Source: BMC Bioinformatics. 2010 May 18;11:257. doi: 10.1186/1471-2105-11-257 (PMC2885376; doi:10.1186/1471-2105-11-257)
Supplement: Additional file 3 — GEO sample and platform accession numbers. This file contains GEO accession numbers and the tissue information used to compare our results with those of Arora and Simpson. [file 1471-2105-11-257-S3.DOC]

| **GEO series accession** | **Platform** | **GEO sample accession** | **Tissue** |
| --- | --- | --- | --- |
| GSE3526 | GPL570 | GSM80654.CEL | Heart Atrium |
| GSE3526 | GPL570 | GSM80655.CEL | Heart Atrium |
| GSE3526 | GPL570 | GSM80656.CEL | Heart Atrium |
| GSE3526 | GPL570 | GSM80700.CEL | Midbrain |
| GSE3526 | GPL570 | GSM80701.CEL | Midbrain |
| GSE3526 | GPL570 | GSM80702.CEL | Midbrain |
| GSE3526 | GPL570 | GSM80703.CEL | Midbrain |
| GSE3526 | GPL570 | GSM80704.CEL | Midbrain |
| GSE3526 | GPL570 | GSM80705.CEL | Midbrain |
| GSE3526 | GPL570 | GSM80706.CEL | Midbrain |
| GSE3526 | GPL570 | GSM80707.CEL | Lung |
| GSE3526 | GPL570 | GSM80710.CEL | Lung |
| GSE3526 | GPL570 | GSM80712.CEL | Lung |
| GSE3526 | GPL570 | GSM80728.CEL | Liver |
| GSE3526 | GPL570 | GSM80729.CEL | Liver |
| GSE3526 | GPL570 | GSM80730.CEL | Liver |
| GSE3526 | GPL570 | GSM80731.CEL | Kidney medulla |
| GSE3526 | GPL570 | GSM80732.CEL | Kidney medulla |
| GSE3526 | GPL570 | GSM80733.CEL | Kidney medulla |
| GSE3526 | GPL570 | GSM80734.CEL | Kidney medulla |
| GSE3526 | GPL570 | GSM80757.CEL | Ovary |
| GSE3526 | GPL570 | GSM80758.CEL | Ovary |
| GSE3526 | GPL570 | GSM80759.CEL | Ovary |
| GSE3526 | GPL570 | GSM80790.CEL | Skeletal Muscle |
| GSE3526 | GPL570 | GSM80791.CEL | Skeletal Muscle |
| GSE3526 | GPL570 | GSM80792.CEL | Skeletal Muscle |
| GSE3526 | GPL570 | GSM80853.CEL | Testes |
| GSE3526 | GPL570 | GSM80868.CEL | Testes |
| GSE3526 | GPL570 | GSM80869.CEL | Testes |
